# Thyroid Cancer Presenting as Aspiration Pneumonia: A Tale of Retrosternal Goiters

**DOI:** 10.7759/cureus.35861

**Published:** 2023-03-07

**Authors:** Muhammad Asif, Wahab J Khan, Sadia Aslam, Ifrah Nadeem, Anthony Hericks

**Affiliations:** 1 Internal Medicine, Avera McKennan Hospital and University Health Center, Sioux Falls, USA; 2 Internal Medicine, University of South Dakota Sanford School of Medicine, Sioux Falls, USA; 3 Pulmonary and Critical Care Medicine, Avera McKennan Hospital and University Health Center, Sioux Falls, USA

**Keywords:** dysphagia surgery, ectopic thyroid mass, retrosternal goiter, aspiration pneumonia, mediastinal mass

## Abstract

Abnormal enlargement of the thyroid gland is called goiter. Structurally, it can be nodular or diffuse. Usually, it presents as an anterior cervical mass; however, less commonly, it presents as a retrosternal mass causing symptoms of compression on the surrounding structures. Most patients with goiter are asymptomatic due to the euthyroid nature of the disease. However, sometimes they can be hypo or hyperthyroid depending on the etiology of the goiter. Here, we present the case of a patient without any previously known goiter who presented to the hospital with shortness of breath and was found to have hypoxic respiratory failure as his first noticed sign of thyroid disease. Diagnostic workup revealed retrosternal goiter causing compression effect on the esophagus and trachea resulting in dysphagia and aspiration. The patient was treated with feeding tube placement, followed by surgical resection of the mediastinal mass.

## Introduction

Abnormal enlargement of the thyroid gland is called goiter. Structurally, it can be nodular or diffuse. By location, it usually presents as an anterior cervical mass; however, less commonly, it presents as a retrosternal mass causing symptoms of compression on the surrounding viscera [1]. Most patients with goiter are asymptomatic due to the euthyroid nature of the disease; however, sometimes they can be hypo or hyperthyroid depending on the etiology of goiter. Here, we present a case where a patient without any known thyroid history was admitted to the hospital with shortness of breath, hypoxic respiratory failure, and aspiration pneumonia as his first noticed sign of thyroid disease.

## Case presentation

**Figure 1:**
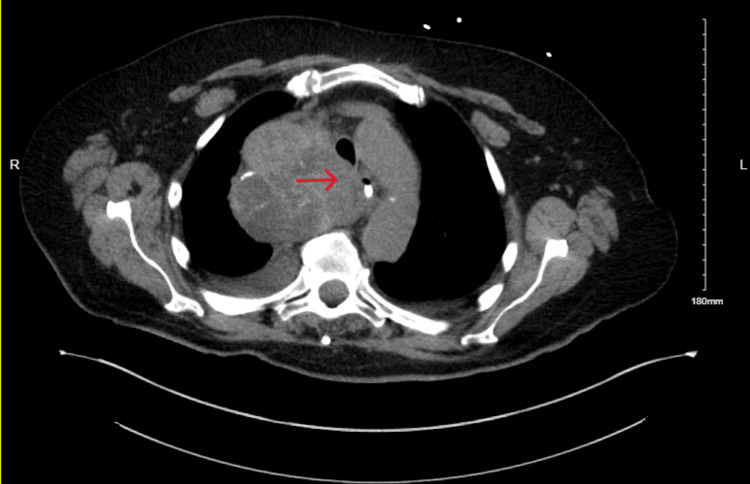
Mediastinal mass seen on the CT scan of the chest causing compression of the esophagus and trachea.

A 68-year-old male presented with shortness of breath, generalized weakness, and frequent falls to the emergency room after a recently diagnosed respiratory syncytial virus (RSV) infection. His initial vital signs showed oxygen saturation of 79% on room air, respiratory rate of 28 breaths/minute, heart rate of 78 beats/minute, a temperature of 101.6°F, and blood pressure of 94/48 mmHg. He was placed on supplemental oxygen initially via a regular nasal cannula and then transitioned to a high-flow nasal cannula requiring up to 50 L/minute with 80% FiO_2_. Chest auscultation was noticeable for bilateral rhonchi and coarse crackles at the bases. Mild non-tender nodularity was noted on the thyroid palpation. Chest X-ray showed bilateral airspace opacities. A chest CT scan revealed a large upper mediastinal mass displacing the trachea and esophagus to the right (Figure 1), a large nodule in the left lobe of the thyroid, subcarinal and right hilar lymphadenopathy, and bilateral dependent consolidative opacities, suggesting aspiration pneumonia.

**Figure 2:**
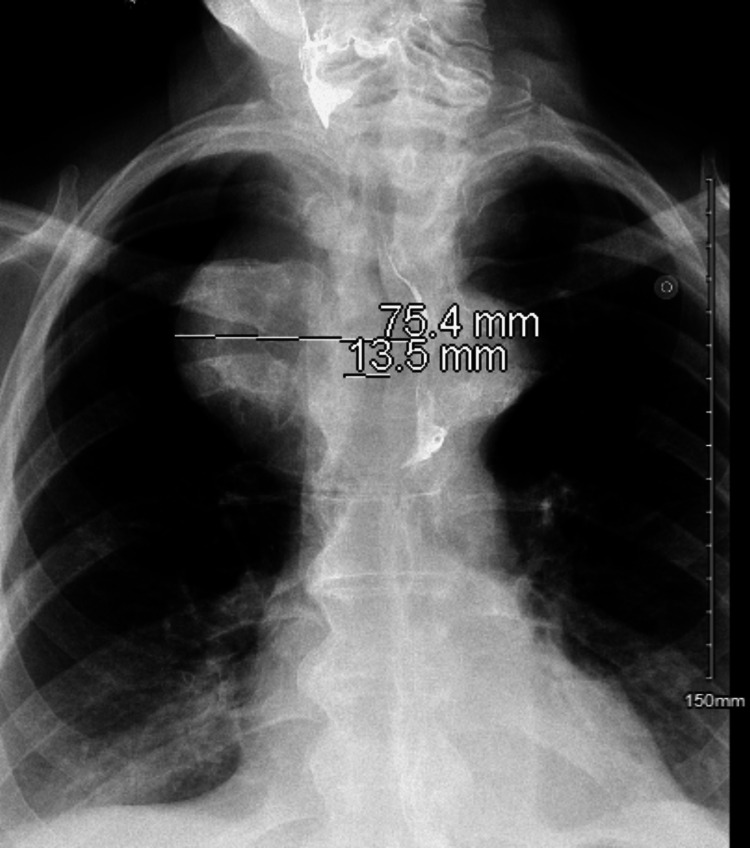
Esophagogram showing a mediastinal mass with compression effect on the esophagus and trachea.

**Figure 3:**
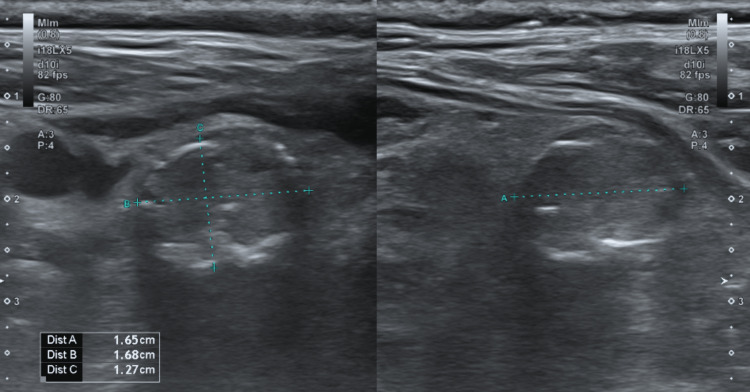
Thyroid nodules seen on an ultrasound of the thyroid.

There was no evidence of pulmonary embolism. A barium esophagogram showed a 7.5 cm mediastinal mass causing 50% narrowing and displacement of the esophagus and compression with a mild left-sided deviation of the trachea (Figure 2). In the barium swallow study, the patient was found to have aspiration with all administered food types due to an abnormal swallowing mechanism. The patient was changed to nothing to eat by mouth, and a nasogastric tube was placed to allow access to nutrition. Additional diagnostic workup included a bronchoscopy with endobronchial ultrasound-guided (EBUS) biopsy of a station 4 lymph node/the mass. The pathology was non-diagnostic and did not find any malignant cells. However, due to high clinical suspicion of a malignant process, the patient underwent a CT-guided biopsy of the mediastinal mass that showed bland thyroid tissue. Ultrasound of the thyroid revealed multiple right and left thyroid nodules (Figure 3). Thyroid-stimulating hormone, free T4, and calcitonin were within normal limits. The patient’s hypoxia and fever resolved within a few days with aspiration precaution and treatment for aspiration pneumonia. Due to persistent dysphagia, a feeding tube was placed. He was stable and discharged from the hospital to a long-term care facility.

On follow-up, the patient underwent an ultrasound-guided fine-needle biopsy of one right-sided and two left-sided thyroid nodules resulting in papillary thyroid cancer. He was referred for surgical evaluation and underwent a successful right-sided thoracotomy with the removal of a grapefruit-sized thyroid mass. The patient is following up with endocrinology as an outpatient for further treatment and surveillance of papillary thyroid cancer. He is also being considered for radioactive iodine therapy.

## Discussion

There is a wide spectrum of differential diagnoses of a mediastinal mass. It includes thymic growth, germ cell tumor, carcinoma, lymphoma, infectious, inflammatory, or malignant lymphadenopathy, meningocele, and other primary or metastatic lesions. The evaluation consists of a thorough history, physical examination, and diagnostic testing, which may include thyroid function tests, imaging of the neck and/or chest with ultrasound and/or CT scans, and tissue sampling (fine-needle biopsy, bronchoscopy with EBUS, mediastinoscopy, or surgical biopsy) in select cases. The thyroid is bordered by the trachea and esophagus posteriorly and the carotid sheath laterally. Enlarging thyroid lobes usually grow anteriorly in front of the neck. As a result of this outward growth, even very large goiters rarely compress the trachea or impinge on the great vessels lateral to the lobes. The trachea, esophagus, blood vessels, and nerves traverse the thoracic inlet. Sometimes thyroid tissue can grow into the thoracic cavity, which can obstruct any entering or exiting structures within the thoracic cavity and cause symptoms of dyspnea, impaired venous drainage, thrombosis, hoarseness of the voice, or dysphagia, as in our patient’s case. Such goiters are called retrosternal. The treatment of retrosternal goiters with or without clinical symptoms is mainly surgical with subtotal or total thyroidectomy [2]. Most substernal goiters grow into the anterior mediastinum and are successfully resected by cervical incision. However, a small portion may require additional incision by sternotomy or thoracotomy, primarily when it extends into the posterior mediastinum, which is not common [3,4]. When goiter presents as airway compression, the outcomes after surgical decompression are very reassuring, as described in one study involving 120 patients who underwent surgery without any significant morbidity or mortality (no death). Only two patients required tracheostomies [4]. The incidence of thyroid cancers in multinodular goiters is widely variable in studies, ranging from 3% to 35%. The most common type is papillary thyroid cancer, and when combined with follicular cancer, it constitutes 97% of thyroid cancers that are managed similarly. Surgical thyroid lobectomy or total thyroidectomy is followed by radioactive iodine therapy. Patients usually undergo surveillance by an endocrinologist with routine ultrasound imaging and thyroglobulin level monitoring. Many patients require replacement or suppressive levothyroxine following the initial treatment. The prognosis of differentiated thyroid cancers is good, and most treated patients with papillary cancer do not die of their disease. Compared with papillary thyroid cancer, follicular cancer typically occurs in older patients. In addition, follicular cancer is more commonly associated with an aggressive clinical course, distant metastases, and higher mortality than papillary thyroid cancer. Women may have a better prognosis than men [5,6].

## Conclusions

Goiter is usually first noticed as a visible neck mass. However, some patients may present with signs and symptoms of hypo or hyperthyroidism. On the other hand, when it extends to the retrosternal space, obstructive or aspiration pneumonia can be its first presentation. Diagnosing a retrosternal goiter may not be difficult nowadays due to the availability of new imaging modalities and interventions, but underlying tissue diagnosis may be challenging to confirm despite repeated sampling. Therefore, clinicians should have high suspicion, and patients may need multiple biopsies by different methods to rule out any neoplastic processes.
